# Molecular Networks in FGF Signaling: Flotillin-1 and Cbl-Associated Protein Compete for the Binding to Fibroblast Growth Factor Receptor Substrate 2

**DOI:** 10.1371/journal.pone.0029739

**Published:** 2012-01-03

**Authors:** Ana Tomasovic, Stephanie Traub, Ritva Tikkanen

**Affiliations:** 1 Institute of Biochemistry, University of Giessen, Giessen, Germany; 2 Institute of Biochemistry II, University Clinic of Frankfurt, Frankfurt am Main, Germany; University of São Paulo, Brazil

## Abstract

Fibroblast growth factor receptor substrate 2 (FRS2α) is a signaling adaptor protein that regulates downstream signaling of many receptor tyrosine kinases. During signal transduction, FRS2 can be both tyrosine and threonine phosphorylated and forms signaling complexes with other adaptor proteins and tyrosine phosphatases. We have here identified flotillin-1 and the cbl-associated protein/ponsin (CAP) as novel interaction partners of FRS2. Flotillin-1 binds to the phosphotyrosine binding domain (PTB) of FRS2 and competes for the binding with the fibroblast growth factor receptor. Flotillin-1 knockdown results in increased Tyr phosphorylation of FRS2, in line with the inhibition of ERK activity in the absence of flotillin-1. CAP directly interacts with FRS2 by means of its sorbin homology (SoHo) domain, which has previously been shown to interact with flotillin-1. In addition, the third SH3 domain in CAP binds to FRS2. Due to the overlapping binding domains, CAP and flotillin-1 appear to compete for the binding to FRS2. Thus, our results reveal a novel signaling network containing FRS2, CAP and flotillin-1, whose successive interactions are most likely required to regulate receptor tyrosine kinase signaling, especially the mitogen activated protein kinase pathway.

## Introduction

Fibroblast growth factor receptor substrate 2 (FRS2/FRS2α/SNT1) is a membrane linked docking protein originally identified as a protein that becomes tyrosine phosphorylated upon nerve growth factor (NGF) or fibroblast growth factor (FGF) stimulation in PC12 cells [1], [2], [3]. Together with FGF receptor substrate 3 (FRS3/FRS2β/SNT2), it belongs to the FRS adaptor protein family [4]. In this paper, we will use the name FRS2 for FRS2α/SNT1, and FRS3 for FRS2β/SNT2 for the sake of clarity.

FRS2 and FRS3 share a similar structure and 48% of amino acid sequence identity. In the N-terminus, they contain a consensus myristoylation sequence which is important for the membrane localization [1]. This sequence is followed by a phosphotyrosine binding (PTB) domain that is highly similar between the two proteins. The PTB domain binds specific peptides of certain receptor tyrosine kinases (RTKs) with or without tyrosine phosphorylated residues [5], [6].

FRS2 is ubiquitously expressed with the highest expression in brain, kidney, lung, ovary and testis and can be detected at all developmental stages of a mouse [7]. FRS2 knockout mice show embryonic lethality due to severe problems in gastrulation [8], [9], demonstrating how crucial FGF signaling is in animal development. In contrast to FRS2, the expression of FRS3 protein begins around day 9 and is restricted to tissues of neuronal origin [7], [10]. When exogenously expressed in FRS2-null mouse embryonic fibroblasts, FRS3 compensates for the loss of FRS2 by stimulating FGF induced activation of extracellularly regulated kinase (ERK), a member of the mitogen activated protein (MAP) kinase family [10].

Both FRS2 and FRS3 are tyrosine phosphorylated in response to NGF, FGF [9], [11] and glial derived neurotrophic factor (GDNF) [12], [13], but only FRS2 is threonine phosphorylated. Phosphorylation of 8 threonine residues in FRS2 occurs as a response to stimulation with FGF, epidermal growth factor (EGF), insulin and platelet derived growth factor (PDGF). This represents a negative feedback mechanism in which activated ERK inhibits further tyrosine phosphorylation of FRS2 by phosphorylating its threonine residues [14], [15]. FRS2 plays an important role in FGF dependent proliferation and migration of the cells [1] and in differentiation of PC12 cells by regulating sustained ERK activity upon FGF or NGF stimuli [1], [16], [17]. It is tyrosine phosphorylated upon insulin treatment, but its precise role in insulin signaling pathway remains largely unknown [18].

The C-terminus of FRS proteins bears multiple tyrosine phosphorylation sites (6 Tyr in FRS2), which, when phosphorylated by specific RTKs, recruit SH2-domain containing proteins such as adaptor protein Grb2 (4 Tyr in FRS2) and protein tyrosine phosphatase Shp2 (2 Tyr in FRS2) [1], [19]. The recruitment of Grb2 eventually results in a strong activation of PI3-kinase signaling and moderate activation of ERK pathway [9], [20], while phosphorylation and subsequent activation of Shp2 will result in a strong activation of ERK signaling [9].

The two members of the flotillin/reggie protein family, flotillin-1/reggie-2 (flot-1) and flotillin-2/reggie-1 (flot-2) are associated with specific membrane microdomains enriched in cholesterol and sphingolipids, also called rafts (For a review, see [21], [22]). Their membrane association is mediated by palmitoylation (both flot-1 and flot-2) and myristoylation (flot-2 only), but neither protein contains a transmembrane domain [23], [24]. Flotillins are widely expressed and well conserved between species, but their molecular function has remained somewhat enigmatic. Flotillins have been shown to participate in various signaling processes, including insulin and EGF receptor signaling [25], [26], [27], [28], in endocytosis [29], phagocytosis [30] and cell adhesion [27]. In addition, a role in neuronal regeneration has been suggested in goldfish and zebrafish [31], [32], but the evidence for such a role in mammalian animal models is lacking. Our earlier results show that flot-2 becomes Tyr phosphorylated by Src kinases and is endocytosed together with flot-1 in EGF stimulated cells [25], [27].

Although functional implications for flotillins in diverse cellular processes have been accumulating, in many cases the exact molecular mechanisms of flotillin action and the interaction partners remain to be identified. One of the known interaction partners of flotillins is the Cbl-associated protein (CAP), also known as Ponsin, which is an adaptor protein containing multiple SH3 (Src homology 3) domains and one or two sorbin homology (SoHo) domains [33]. During insulin receptor signaling, CAP is responsible for recruiting the ubiquitin ligase Cbl into membrane rafts by means of interacting with flotillin-1 [26]. Furthermore, CAP has been shown to be localized in focal adhesions and to mediate processes requiring actin remodeling [34]. Our recent findings also suggest that CAP is involved in signaling and becomes phosphorylated by the Abl kinase [35].

Here, we have identified the PTB-domain containing adaptor proteins FRS2 and FRS3 as novel interaction partners of flot-1. Membrane association and cellular localization of FRS2 was shown to be partly dependent on flot-1. We observed an interaction between flot-1 and FRS2 in mouse tissues. Intriguingly, FRS2 appears to be more Tyr phosphorylated in flot-1 knockdown cells, indicating that the interaction with flot-1 regulates the downstream signaling of FRS2. Moreover, since flot-1 binds to the PTB domain of FRS2, it competes for the binding with the FGF receptor. Furthermore, flot-1 is necessary for the signaling induced recruitment of FRS2 into lipid rafts. In addition to flot-1, FRS2 was also found to interact with CAP, which has been linked to flot-1 during insulin signaling [26]. The binding domains of these three proteins overlap, and thus flot-1 and CAP compete for the binding to FRS2. Here, we have dissected the putative role of these proteins during receptor tyrosine kinase signaling.

## Results

**Figure 1:**
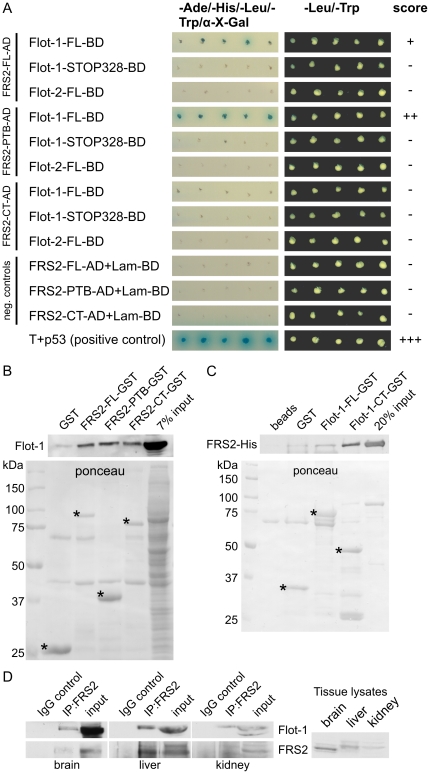
Identification of interaction domains in FRS2 and flot-1. (A) Yeast two-hybrid analysis of the interaction between FRS2 and flot-1 domains. Interaction is indicated as growth of blue colonies on nutrient deficient plates containing α-X-galactoside. (B) FRS2 domains were produced as GST fusion proteins, immobilized on glutathione beads and tested for the interaction with endogenous flot-1 from HeLa cell lysates. Upper blot: detection of bound flot-1, lower blot: ponceau staining of the respective GST proteins. (C) FL flot-1 or its C-terminal half were produced as GST fusion proteins, immobilized on beads and incubated with purified FRS2-His. Upper blot: detection of bound FRS2-His, lower blot: ponceau staining of the purified GST proteins. Specific bands for the GST fusions are marked with *. (D) FRS2 was immunoprecipitated from lysates of 25 mg of mouse tissue (brain, liver and kidney), and the coprecipitation of flot-1 was studied. Antibody against flag tag (IgG control) was used as a control for the immunoprecipitation. Right part shows a blot for FRS2 with total tissue lysates (equal total protein amount).

In search of novel interaction partners of flotillins, we performed a yeast two-hybrid (Y2H) screen of a human brain library using rat flot-1 as a bait, in which several clones containing a partial sequence of FRS3 were obtained (data not shown). Since FRS3 shows a high homology to the more ubiquitously expressed FRS2, we tested the possibility of a direct interaction of flot-1 with FRS2 and characterized the interacting domains in a Y2H assay (Fig. 1A). The constructs used for this are shown in Suppl. Fig. S1A. Interaction was detected as growth of the transformed yeast colonies on nutrient deficient plates and blue colour (Fig. 1A). Full-length (FL) flot-1 was found to interact with the FL FRS2 and the PTB domain of FRS2, whereas no interaction was detected with the C-terminal part of FRS2. Interestingly, flot-2 did not interact with any of the FRS2 fragments. We also attempted to produce deletion fragments of flot-1 (see Suppl. Fig. S1A), but the C-terminal parts flot-1-CT (amino acids 226–428) and flot-1-CC (327–428) displayed autoactivity, resulting in blue colonies without any interaction partner. Furthermore, flot-1-NT (amino acids 1–253) was poorly expressed, preventing any conclusions about its interaction with FRS2. However, a deletion mutant of flot-1 missing 100 amino acids from the C-terminal end (Flot-1-STOP328) was unable to interact with any of the FRS2 fragments, indicating that the interaction domain may reside in the far C-terminus of flot-1. Suppl. Fig. S1B shows the expression in the yeast of the constructs used in Fig. 1A and a test for autoactivation in the yeast.

We next used GST-pulldown analysis to verify the interaction between FRS2 and flot-1 (Fig. 1B–C). FL FRS2, the PTB and CT domains were expressed as GST fusions and immobilized on glutathione beads (Fig. 1B). All three variants of FRS2 were capable of binding substantial amounts of flot-1 from HeLa cell lysates. To demonstrate a direct interaction, we used bacterially expressed, purified proteins. FL Flot-1-GST and Flot-1-CT-GST were immobilized on sepharose beads and tested for their binding to His-tagged FRS2 (Fig. 1C). FRS2 was clearly bound by FL and even more by the CT of flot-1. Thus, our yeast two-hybrid and pulldown data suggest that FRS2 interacts with flot-1 but not with flot-2, and the interaction involves both the PTB domain and C-terminus of FRS2 and the C-terminal part of flot-1.

To verify the interaction between flot-1 and FRS2 in vivo, we performed coimmunoprecipitation from mouse tissues (Fig. 1D). FRS2 was immunoprecipitated using same amounts of homogenized mouse tissues which express the highest amounts of FRS2 (brain, liver and kidney; see right part of Fig. 1D for relative expression levels). Coimmunoprecipitation of flot-1 with FRS2 was seen from all three tissues, whereas no precipitation of flot-1 was detected when an isotype-matched control antibody was used.

**Figure 2:**
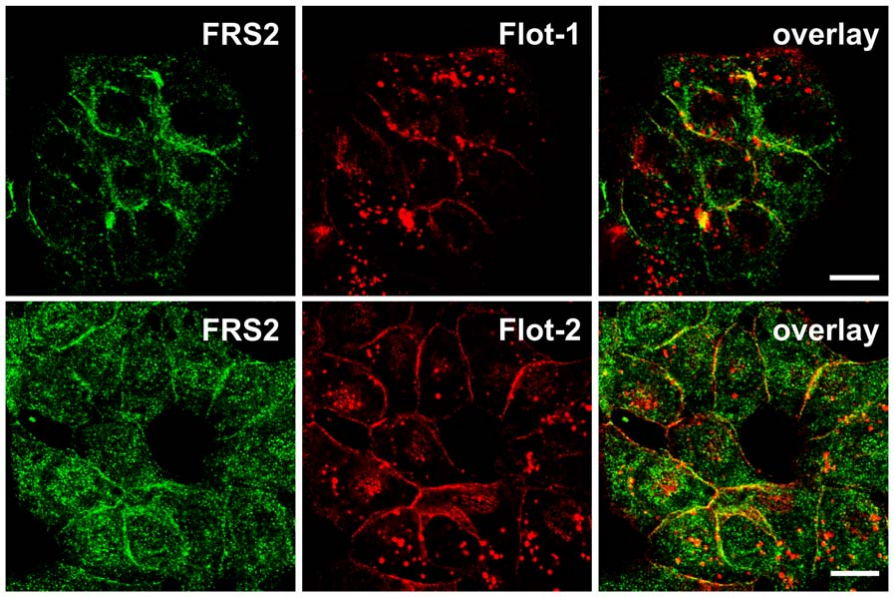
Endogenous flot-1 and flot-2 colocalize with FRS2 in Hep3B cells. Cells were grown in a medium containing FCS and stained with antibodies against endogenous FRS2 (green) and flot-1 or flot-2 (red). Scale bars 10 µm.

To study the possible colocalization of flot-1 with FRS2 in animal cells, we chose Hep3B cells which express high amounts of endogenous FRS2 and flotillins. Immunofluorescence staining of FRS2 and flotillins revealed that in serum-grown Hep3B cells, FRS2 was mainly localized at the plasma membrane with some diffuse and vesicle-like staining in the cytoplasm (Fig. 2). Flot-1 (Fig. 2, upper row) and flot-2 (Fig. 2, lower row) were both detected at the plasma membrane, where they colocalized with FRS2. However, little if any colocalization was detected in the endosomes, which contained high amounts of flotillins.

**Figure 3:**
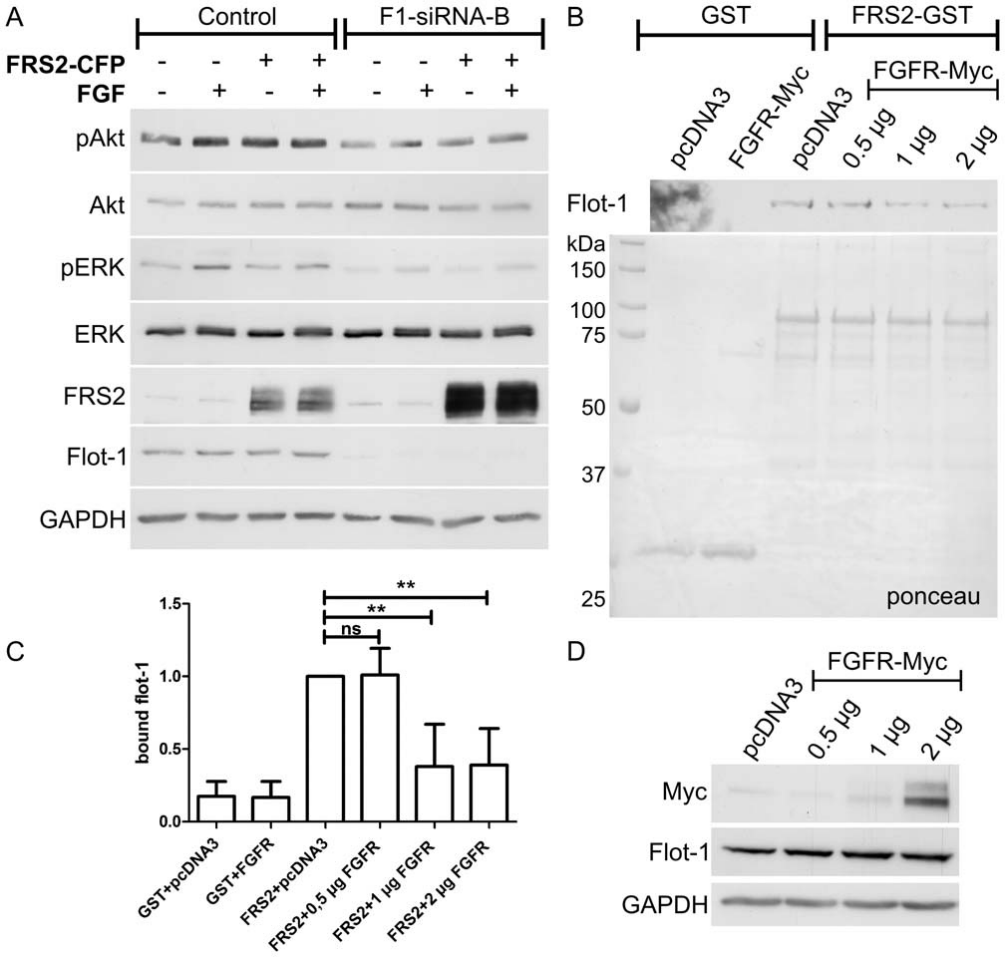
Overexpression of FRS2 does not compensate for the signaling defects in flot-1 knockdown cells. FGF receptor and flot-1 compete for the binding to FRS2. (A) Flot-1 was knocked down in HeLa cells by means of siRNAs and the cells were transfected with FRS2-CFP. Starved cells were stimulated with FGF for 5 min, and the activation of Akt (uppermost blot) and ERK2 (3^rd^ blot) was measured with phospho-specific antibodies. The third blot from the bottom shows the analysis of the transfection efficiency of FRS2-CFP and of the 2^nd^ one the knockdown efficiency of flot-1. Lowermost blot (GAPDH) shows equal protein loading. (B) Purified FRS2-GST was immobilized on sepharose and incubated with lysates of HeLa cells transfected with increasing amounts of FGFR-myc (0.5 to 2 µg). The binding of endogenous flot-1 from these lysates was tested (upper blot). (C) Quantification of the flot-1 bound to FRS2. In the presence of increasing amounts of FGFR, the binding is significantly reduced. (D) Expression of FGFR was verified by Western blot.

Our previous findings have shown that upon knockdown of flot-1 in HeLa cells, EGF-stimulated activation of MAP kinase signaling is inhibited (Amaddii et al., under revision). Furthermore, it has been shown that overexpression of FRS2 results in a dose-dependent enhancement of ERK activation upon EGF signaling [15]. Thus, we tested if overexpression of FRS2 would be able to compensate for the MAP kinase activation defects upon receptor tyrosine kinase stimulation in flot-1 knockdown cells. However, since the best characterized function of FRS2 lies within FGF receptor (FGFR) signaling, where it also mediates an increase of MAP kinase activation, we here used FGF stimulation instead of EGF. Phosphorylation of Akt and ERK2 after 5 min of FGF stimulation were found to be reduced in flot-1 knockdown cells as compared to the control cells (Fig. 3A), consistent with our previous findings (Amaddii et al., under revision). Overexpression of FRS2-CFP did not result in correction of the diminished phosphorylation of these proteins after FGF stimulation in flot-1 knockdown cells. Expression of FRS2-CFP and the knockdown efficiency of flot-1 were verified by means of Western Blot (Fig. 3A). We observed a considerable shift in the gel motility of FRS2 after FGF treatment, most likely due to phosphorylation, in both control and flot-1 knockdown cells. Thus, although the growth factor induced phosphorylation of FRS2 appears to take place in flot-1 knockdown cells, FRS2 is not able to compensate for the observed downstream signaling defects.

FRS2 binds to FGFR cytoplasmic domain by means of its PTB domain but independently of Tyr phosphorylation [6]. Since we could show that this domain also at least partly mediates the interaction to flot-1, we tested if FGFR and flot-1 would compete for the binding to FRS2 (Fig. 3B). For this, we used purified FRS2-GST immobilized on beads and lysates of Hela cells transfected with increasing amounts of myc-tagged FGFR (0.5–2 µg of DNA used for transfection, Fig. 3D). As a transfection control, the empty vector pcDNA3 was used. The binding of endogenous flot-1 to FRS2-GST in the presence of FGFR was found to decrease the more FGFR was present in the lysates (Fig. 3B), but we were unable to obtain a full loss of flot-1 binding by FRS2 due to FGFR. Significantly reduced binding of flot-1 to FRS2 was observed upon increased expression of FGFR (Fig. 3C).

For studies of the function of endogenous FRS2, which is expressed in HeLa cells only in very minor amounts, in the absence of flot-1, we generated stable Hep3B clones in which flot-2 or flot-1 were knocked-down using lentivirus-mediated RNA interference. It has to be noted that knockdown of flot-2 results in a concomitant destabilization and downregulation of flot-1 at the protein level, whereas flot-1 depletion only mildly affects flot-2 expression (Suppl. Fig. S1C), as shown by us and others for various cell lines [25], [36]. Suppl. Fig. S1D shows that very little flot-2 or flot-1 could be detected by means of immunofluorescence staining in the knockdown cells, demonstrating that a high degree of depletion of flotillins was achieved in virtually all cells of the respective clone.

**Figure 4:**
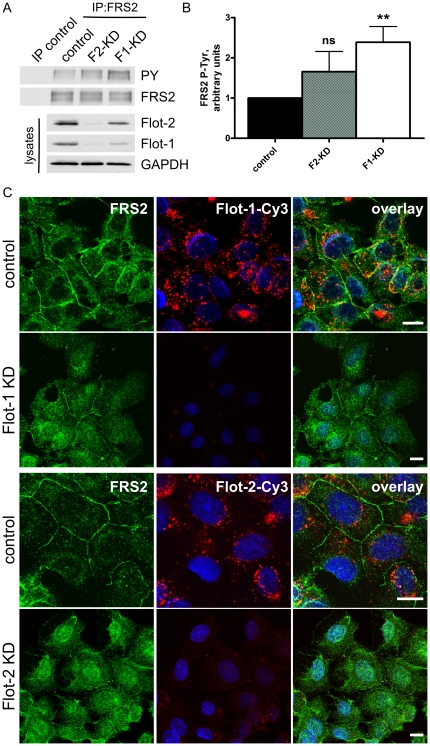
Increased Tyr phosphorylation and solubility of FRS2 in flotillin knockdown cells. (A) FRS2 was immunoprecipitated from serum grown Hep3B cells. The Tyr phosphorylation of FRS2 was measured by means of phospho-Tyr antibodies and found to be increased both in flot-1 and flot-2 knockdown cells. (B) Densitometric quantification of FRS2 phosphorylation with SD (5 independent experiments). F1-KD cells display a significantly increased P-Tyr of FRS2. (C) Hep3B cells were grown under serum, fixed and stained with antibodies against FRS2 (left column) and flotillins (middle). In control cells, FRS2 was localized at the plasma membrane and within the cytosol, whereas in flot-1 or flot-2 knockdown cells, a cytosolic staining was evident. In addition, especially in flot-2 knockdown, some nuclear staining was observed. Right column: overlay with DAPI staining. Scale bars 10 µm.

To see if knockdown of flotillins affects the phosphorylation status of FRS2, we used the stable Hep3B knockdown cells. FRS2 was immunoprecipitated from serum-grown cells, and the blots were probed with anti-phospho-Tyr antibodies (Fig. 4A). Interestingly, we could detect a tendency to increased Tyr phosphorylation of FRS2 in flot-2 knockdown cells and a significantly increased phosphorylation in the flot-1 knockdown cells (Fig. 4B). Since Tyr and Thr phosphorylation of FRS2 have been described to be reciprocally regulated [14], [15], one would expect that the increased Tyr phosphorylation would result in decreased Thr phosphorylation of FRS2 in flotillin knockdown cells. We thus aimed at detecting the Thr phosphorylation of FRS2, which is mediated by ERK, by means of antibodies directed against phosphorylated recognition sites of the ERK kinase. Unfortunately, although a tendency to decreased Thr phosphorylation of FRS2 was indeed visible after depletion of flot-2 or flot-1, the signals were generally very weak and did not allow for any definite conclusions (Data not shown).

FRS2 has been described to associate with cellular membranes by means of myristoylation. Since myristoylation alone can only provide a weak membrane attachment [37], we studied by means of immunofluorescence studies if the cellular localization of FRS2 might depend on flotillins. Although FRS2 was localized at the plasma membrane in the control cells, knockdown of flot-1 or flot-2 resulted in a slight increase in the cytoplasmic staining and some loss of membrane localization of FRS2 (Fig. 4C), indicating that in the absence of flotillins, FRS2 is less membrane associated. In addition, some nuclear staining for FRS2 was detected in flot knockdown cells but not in the control. These results suggest that the membrane association of FRS2 may be facilitated by the presence of flotillins. Since flot-2 and flot-1 knockdown cells gave virtually identical results in these assays, it is likely that the changes in FRS2 membrane association are more attributable to the absence of flot-1 expression, considering that flot-2 knockdown cells also lack flot-1 (Suppl. Fig. S1B).

**Figure 5:**
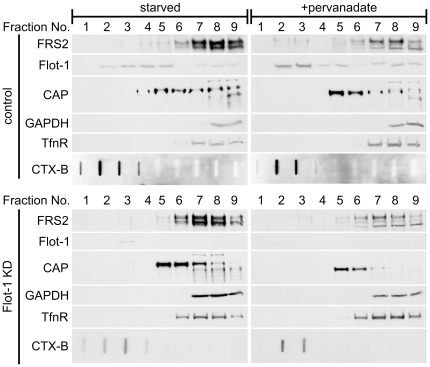
Flotillin-1 is required for the recruitment of FRS2 into light membranes in pervanadate treated cells. Hep3B cells (control: upper panels, flot-1 knockdown: lower panels) were starved overnight and then stimulated with pervanadate. Detergent resistant light membranes were prepared using density gradient centrifugation and found in fractions 1–3 of the gradient. The localization of FRS2, flot-1 and CAP was analyzed. Western blots for transferrin receptor (TfnR), GAPDH and GM1-bound cholera toxin subunit B (CTX-B) were used to control the gradient.

Previous data have shown that in some cell types, FRS2 is constitutively associated with membrane rafts [38], [39]. Thus, the interaction between flot-1 and FRS2 might facilitate the localization of FRS2 into these domains. To test this, we again used the Hep3B cells which were either serum starved or starved and then stimulated with pervanadate to enhance Tyr phosphorylation. Detergent resistant membranes (referred to as rafts below) were isolated using density gradients after detergent extraction [40]. In these gradients, rafts are mainly present in fractions 1–3, as evidenced by the enrichment of cholera toxin in these fractions (Fig. 5). Flot-1 was found to reside in fractions 2–5 in starved cells, whereas in pervanadate stimulated cells, a shift towards raft fractions was observed (Fig. 5, upper part). We were not able to detect FRS2 in the raft fractions in starved cells, whereas in pervanadate stimulated cells, some FRS2 was found in fractions 2–3 together with flot-1. Intriguingly, pervanadate did not induce a shift of FRS2 towards raft fractions in flot-1 knockdown cells (Fig. 5, lower part), implicating that flot-1 is necessary for the stimulation induced recruitment of FRS2 into detergent insoluble membranes in these cells. However, only a fraction of FRS2 becomes raft associated during signaling, contrary to previous findings from other cell types [38], [39].

Flot-1 has previously been described to interact with Cbl-associated protein (CAP). We thus tested in our raft fractions if the localization of CAP would be altered after flot-1 knockdown. In control cells, CAP displayed a broad distribution in the heavier fractions, without localizing to rafts. Although CAP underwent a shift towards lighter membranes upon pervanadate stimulation, it remained concentrated in fractions 5 and 6, without localizing to rafts (Fig. 5, upper part). In flot-1 knockdown cells, CAP was found to reside in fractions 5 and 6 both in starved and stimulated cells (Fig. 5, lower part). Thus, although CAP exhibits a flot-1 dependent localization in these gradients, flot-1 does not recruit CAP into rafts.

**Figure 6:**
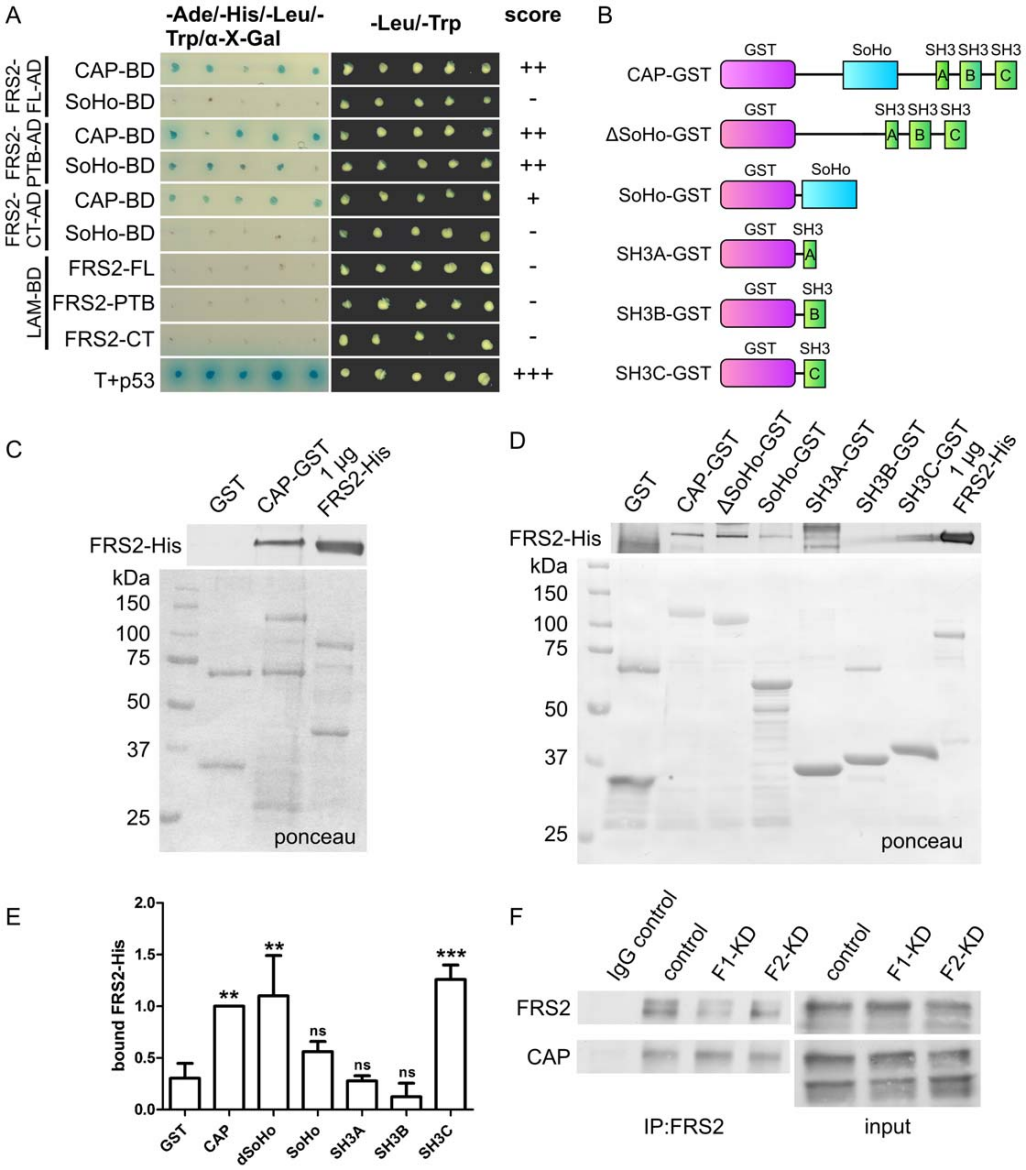
FRS2 directly interacts with Cbl-associated protein. (A) Yeast two-hybrid analysis of the interaction between FRS2 and CAP domains. (B) Structure of the CAP-GST constructs used. (C) and (D) Interaction of purified FRS2-His and CAP-GST proteins. CAP-GST fusion proteins were immobilized on sepharose and tested for the binding of purified FRS2-His. Upper blot shows the bound FRS2-His (anti-His antibody), lower blot the ponceau staining of the GST proteins. 1 µg of FRS2-His was used as a positive control. (E) Quantification of the binding of FRS2 to various CAP domains. A binding of FRS2 significantly higher than background was seen with the full-length CAP, delta-SoHo and the third SH3 domain. (F) Endogenous FRS2 was immunoprecipitated from Hep3B cells, and the binding of endogenous CAP was tested. Please note that several isoforms of CAP are present in Hep3B cells, of which only one appears to bind FRS2.

Previous findings have shown that during signaling of the neurotrophic receptor TrkA, flot-1, FRS2 and CAP all localized into rafts [38]. However, a direct molecular connection between these three proteins has so far not been established. Since flot-1 interacts with both FRS2 and CAP, we tested if FRS2 would be capable of interacting with CAP. Our Y2H analysis showed that FL CAP indeed interacted with FL, PTB and CT domain of FRS2, whereas the sorbin homology (SoHo) domain of CAP only showed an interaction with FRS2 PTB domain (Fig. 6A). Since both FRS2 and CAP posses multiple domains that mediate protein-protein interactions and both CT and PTB in FRS2 appeared to interact with CAP, we used GST pulldown to characterize the interaction domains more closely. Various GST fusion proteins containing CAP domains were generated (Fig. 6B). The purified GST proteins were incubated with purified FRS2-His. A strong interaction was seen between FL proteins (Fig. 6C and D). Interestingly, a CAP mutant lacking the SoHo domain (ΔSoHo) showed a similar binding to FRS2 as the FL CAP, whereas the SoHo-GST interacted less strongly (Fig. 6D). Of the three SH3 domains (src-homology domain 3) of CAP, only the third one (SH3C) was found to bind FRS2. Quantification of the binding showed that full-length CAP, ΔSoHo and SH3C all displayed a significant binding to FRS2 (Fig. 6E). These results imply that multiple domains in FRS2 and CAP are involved in their interaction. These in vitro results were verified by coimmunoprecipitation of the endogenous proteins from Hep3B cells, in which a strong coprecipitation was detected. A similar degree of coprecipitation was detected in control and flot-1 or flot-2 knockdown cells (Fig. 6F), although the coprecipitation may even be slightly increased in flot-1 knockdown cells.

**Figure 7:**
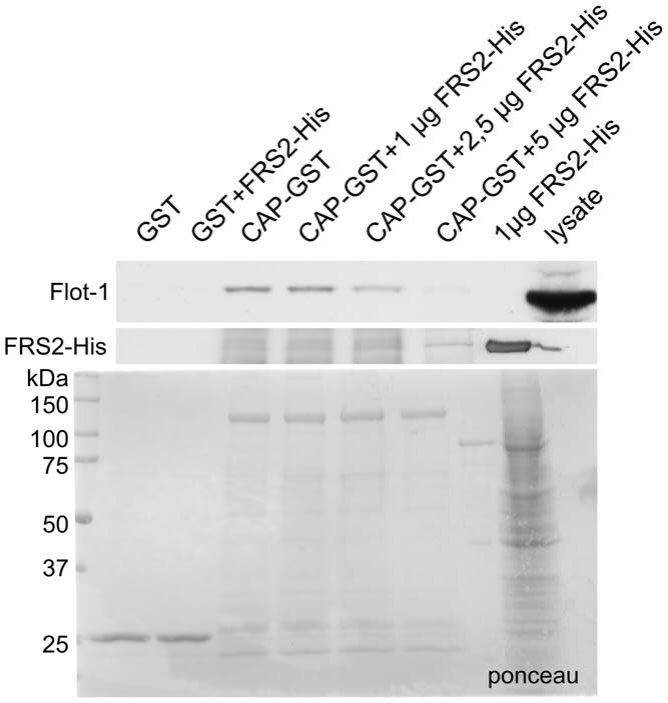
Flot-1 and CAP compete for the binding to FRS2. CAP-GST was immobilized to sepharose and incubated with HeLa cell lysates in the presence of increasing amounts (1–5 µg) of purified FRS2-His. The binding of endogenous flot-1 from the lysates was analyzed by Western blot (upper blot). Middle panel shows the blot for FRS2-His and the lowermost one a ponceau staining of the GST proteins.

Since the SoHo domain of CAP and the PTB domain of FRS2, which were found to interact, are the domains that also mediate the interaction of the respective proteins with flot-1, we tested the hypothesis that CAP and flot-1 might compete for the binding to FRS2. CAP-GST was immobilized on sepharose beads and incubated in the presence of increasing concentrations of purified FRS2-His, and the binding of endogenous flot-1 from HeLa lysates was tested (Fig. 7). The presence of increasing concentrations of FRS2-His indeed was capable of abolishing the interaction of flot-1 with CAP, demonstrating that rather than forming a trimeric complex, FRS2, flot-1 and CAP may compete for the binding of each other due to their partly overlapping interaction domains. Since the amount of FRS2 bound by CAP only modestly increases upon incubation with higher amounts of purified FRS2, whereas CAP-bound flot-1 is clearly reduced, FRS2 most likely binds to flot-1 in the lysate, prevents it from binding to CAP and keeps it in the soluble fraction.

## Discussion

Here we have shown that the signaling adaptor protein FRS2 directly interacts both *in vivo* and *in vitro* with the membrane raft-associated flot-1. This interaction is mediated by the PTB domain and, to a lesser extent, the C-terminus of FRS2 and by the C-terminus of flot-1. We were able to coprecipitate flot-1 together with FRS2 from mouse tissue lysates, demonstrating that this interaction also takes place *in vivo*. Interestingly, flot-2, which shows a high homology to flot-1 and forms stable oligomeric complexes with it [25], does not appear to directly interact with FRS2. However, coprecipitation of flot-2 with FRS2 was detected from cell lysates, which is probably due to the strong association of flot-2 with flot-1 and not a result of a direct interaction with FRS2.

Further proof for the functional role of the interaction of flot-1 with FRS2 was provided by our results showing that depletion of flot-1 affects the cellular localization of FRS2 in that it appeared to be more soluble. Furthermore, in vanadate stimulated cells, a small fraction of FRS2 was recruited into detergent resistant membranes, which did not take place in the absence of flot-1. Even more importantly, FRS2 was not able to rescue the phosphorylation deficiency of ERK in flot-1 knockdown cells. Overexpression of FRS2 has previously been shown to result in increased ERK activation [15]. If the ERK activation pathways that flot-1 and FRS2 participate in were separate, one would expect that FRS2 overexpression would result in normal ERK phosphorylation. However, if FRS2 and flot-1 are functionally interconnected, FRS2 probably residing upstream of flot-1, no compensation would take place, as observed here. Thus, the interaction of FRS2 and flot-1 during FGFR signaling may be required for a proper activation of MAP kinases.

Flot-1 does not contain any typical PTB domain binding motifs (NPXY or NPXpY) although its interaction with FRS2 is to the most part mediated by the FRS2 PTB domain. Thus, other binding motifs must be responsible for the interaction. Consistently, the PTB domain of FRS2 can bind to both phosphorylated and nonphosphorylated sequences. It has been shown to interact with the Trk A receptor by means of a classical NPXpY motif, whereas its interaction with the juxtamembrane region of FGF receptor 1 involves an amino acid sequence lacking both Tyr and Asn residues [5], [6], [41]. However, Tyr phosphorylation was found to increase the binding of the full-length FRS2 but not of the PTB domain to FGFR1 [42]. We here observed an increased Tyr phosphorylation of FRS2 in the absence of flot-1. Previous studies have shown that Tyr and Thr phosphorylation of FRS2 are reciprocally regulated [14], [15], [39]. Unfortunately, we were not able to measure FRS2 Thr phosphorylation, which is mediated by ERK, but it is likely to be reduced. This may even be a direct consequence of the reduced ERK activity in flot-1 knockdown cells, since inhibition of ERK activity has been shown to result in decreased Thr and concomitantly increased Tyr phosphorylation of FRS2 [15].

Another novel interaction partner of FRS2 discovered in the present study is CAP, an adaptor protein with three SH3 domains. Both the PTB domain and CT of FRS2 were found to mediate this interaction, whereas in CAP, the SoHo domain, together with the third SH3 domain, seems to bind to FRS2. In our GST pulldown assays, deletion of the CAP SoHo domain did not result in a major reduction in binding to FRS2. Furthermore, both the FL CAP and the third SH3 domain showed about similar binding efficiency to FL FRS2, implicating a co-operative mode of binding. SH3 domains bind to proline rich sequences, several of which are present in FRS2, one of them even residing within the PTB sequence [2]. In the Y2H assay, the SoHo domain only bound to the PTB domain, whereas FL CAP bound to FRS2 CT as well. Thus, this would suggest that the SH3 domain of CAP may also be capable of interacting with the poly-Pro sequences in the more C-terminal part of FRS2.

Interestingly, CAP has previously been linked to flot-1 during insulin signaling [26]. Furthermore, it has been postulated that the activated NGF receptor TrkA is translocated into flotillin-containing membrane rafts. Differentiation and neurite growth of PC12 cells can be induced by NGF, which binds to its receptor TrkA and induces a signal transduction through rafts. In PC12 cells, a very transient association of TrkA with CAP, which in turn interacted with flot-1 with similar kinetics, was observed. However, CAP was detected in a constitutive complex with an adaptor protein APS [43], which has been shown to associate only with the activated TrkA [44]. This would suggest that during TrkA signaling, CAP associates with flot-1 and activated TrkA only very shortly, possibly mediating raft recruitment of TrkA, but then dissociates from them. Intriguingly, FRS2 has been shown to reside in the rafts in which the activated TrkA is recruited [38], and it is already long recognized that FRS2 is an important regulator of TrkA signaling [2], [3], [11], [14], [17], [38], [45], and may even participate in insulin signaling [18]. However, FRS2 has never been connected either with flot-1 or CAP before. Since flotillins were originally discovered as neuronal regeneration proteins [32], and knockdown of flotillins in neuronal N2A cells causes reduced neurite outgrowth [31], flotillins might indeed modulate neuronal differentiation together with FRS2 and CAP, most likely by regulating the activation of MAP kinases.

We here show that FRS2 can bind to both flot-1 and CAP, whereas previous studies have shown that CAP and flot-1 also interact with each other, and this interaction also requires the SoHo domain of CAP [26], [33]. The interaction of flot-1 with FRS2 may be required for the recruitment FRS2 to membranes or even membrane rafts, as has been suggested for the interaction of flot-1 with CAP [33]. This might in turn be necessary for a productive interaction of FRS2 with its signaling partners, such as FGF receptor. Although we cannot exclude that a trimeric complex between FRS2, flot-1 and CAP exists in the cells, the overlapping binding domains would rather suggest that these proteins compete for the binding to each other, as we also observed for the binding of FGFR and flot-1 to FRS2. Alternatively, FRS2, CAP and flot-1 may bind to each other in a successive manner, thus regulating the formation of downstream signaling complexes. In line with this, FRS2 has been shown to be connected by means of the growth factor binding protein-2 (Grb2) with the ubiquitin ligase cCbl during FGFR signaling [46]. On the other hand, cCbl regulates insulin receptor signaling by means of its interaction with CAP, which then binds to flot-1 [26]. Thus, the signalosomes that are formed by FRS2, flot-1 and CAP may vary according to the signaling receptor and the respective cellular response. Importantly, strong evidence connects all three proteins to MAPK activation, and FRS2 has been suggested to act as a scaffolder during MAPK signaling [1], [14], [34], [38], [47], [48], [49]. Our unpublished results imply that during EGF receptor signaling, flot-1 is found in complex with RAF, MEK and ERK kinases, suggesting a scaffolding function (Amaddii et al, under revision). Very interestingly, we identified another putative MAP kinase scaffolder, mitogen activated protein kinase organizer-1, MORG1 [50], [51] as a further interaction partner of flot-1 in our yeast two-hybrid screen (our unpublished results). Thus, the most likely mechanism how the interaction of FRS2 with flot-1/CAP modulates signal transduction is the regulation of MAP kinase function and the cellular activities resulting from MAP kinase activity, such as proliferation and differentiation.

## Materials and Methods

### Plasmid constructs

FRS2 cDNA (NM_006654.3) was obtained by reverse transcription using the primers:

5′-CTATAGAATTCATGGGTAGCTGTTGTAGCTG-3′

5′-CTATAAGATCTAGCATGGGCAGATCAGTACTATTG-3′

and cloned into vectors pECFP-N1 or pGADT7 (Clontech). A sequence encoding the PTB domain of FRS2 was generated by PCR using the primers

5′-CTATAGAATTCATGGGTAGCTGTTGTAGCTG-3′

5′-CTATAAGATCTTCATATACTATTATTTTGCATAATCTC-3′

and the C-terminal region with the primers

5′-CTATAGAATTCAATGTGGTGGAAGAGCCAGTTG-3′

5′-CTATAAGATCTAGCATGGGCAGATCAGTACTATTG-3′

GST-fusion constructs of FRS2 were constructed by subcloning into vector pGEX-4T-1 (GE Healthcare), and FRS2-His was obtained by subcloning into pSCherry2 (Eurogentec, Seraing, Belgium). The flot-1 (NM_022701.2) constructs in pGBKT7 vector have been described previously [24], [52]. Rat flotillin-1 in pET41a was generated by PCR cloning. CAP-Flag (murine isoform 1, GenBank accession number: U58883) and SoHo-BD were a kind gift of Dr. A. Saltiel. Other CAP constructs were generated using PCR. FGFR-Myc construct was a kind gift of I. Kovacevic (University of Frankfurt).

### Antibodies

Rabbit polyclonal antibodies against FRS2 (Western blotting) and ERK1/2 and the monoclonal antibody against pERK1/2 were from Santa Cruz Biotechnology (Santa Cruz, CA, USA). Rabbit polyclonal antibodies against Flag tag and FRS2 (for immunoprecipitation and immunofluorescence) were from Sigma-Aldrich (Taufkirchen, Germany). Rabbit polyclonal antibodies against Akt, phospho-Akt (Ser473) and phospho-Tyr were from Cell Signaling Technology (Danvers, MA, USA). Monoclonal antibodies against flot-1 and flot-2 were from Transduction Laboratories (Franklin Lakes, NJ, USA) and anti-CAP was from Upstate (Lake Placid, NY, USA). A mouse monoclonal antibody against GAPDH was from Biozol (Eching, Germany). The primary antibodies used for immunofluorescence were detected with a Cy3-conjugated goat anti-mouse antibody (Jackson Immunoresearch, West Grove, PA, USA) and with an Alexa Fluor 488 donkey anti-rabbit antibody (Molecular Probes, Karlsruhe, Germany). Secondary antibodies goat anti-mouse and goat anti-rabbit coupled to horseradish peroxidase (HRP) were from Southern Biotechnologies (Birmingham, AL, USA) and Zymed (Invitrogen, Karlsruhe, Germany), respectively.

### Yeast-two-hybrid analysis

50 ml YPDA was inoculated with several fresh colonies of the yeast strain AH109, and the culture was incubated with shaking at 30°C for 16 h to stationary phase (OD_600_>1.5). An aliquot of the culture was transferred to 300 ml YPDA medium and incubated at 30°C with shaking until OD_600_ 0.5±0.1. Preparation of competent cells and cotransformation with bait and prey constructs were done according to the manufacturer's protocol (Matchmaker GAL4 Two-Hybrid System 3, Clontech). Transformants were plated on SD/-Leu/-Trp plates for selection. After a few days, colonies were picked and transferred onto SD/-Ade/-His/-Leu/-Trp/X-α-gal plates for the detection of galactosidase activity and onto SD/-Leu/-Trp plates for growth control. Plates were photographed 3 days after plating.

### Preparation of yeast lysates

Yeast cell pellet from a 2.5 ml overnight culture was washed and snap-frozen in liquid nitrogen. The pellets were resuspended in 100 µl cracking buffer (8 M urea, 5% w/v SDS, 40 mM Tris-HCl, 0.1 mM EDTA, 0.4 mg/ml bromphenolblue) supplemented with protease inhibitor cocktail, 5 mM PMSF and 10% β-mercaptoethanol. Thereafter, 100 µl of glass beads were added into the mixture which was vigorously vortexed and incubated for 10 min at 70°C. Insoluble material was pelleted by centrifugation at 21 900 g for 5 min. The supernatant was kept, while the insoluble pellet was further cooked for 5 min and then centrifugated for 5 min at 21 900 g. The supernatants of these two extractions were pooled and analyzed by Western blot.

### Cell culture and transfection

HeLa cells were cultured at 8% CO_2_ and 37°C in Dulbecco's modified Eagle's medium (DMEM, Invitrogen, Karlsruhe, Germany), containing high glucose, 10% fetal calf serum (FCS, Invitrogen), 100 U/ml penicillin and 100 µg/ml streptomycin (Sigma-Aldrich, Taufkirchen, Germany). Hep3B cells were maintained in the same medium but at 5% CO_2_. For transient transfections, Hela cells were seeded out on 6 well plates and grown to a confluency of 90%, after which they were transfected with 1 µg plasmid DNA using Lipofectamine 2000 (Invitrogen).

### GST-protein expression

The following bacterial strains were used for the expression of fusion proteins: BL21 transformed with either pGEX-4T-1 or FRS2-GST constructs and the strain Rosetta transformed with either pET41a, full length CAP-GST, flot-1-GST or one of the deletion constructs of CAP. The bacteria were grown at 37°C until OD_600_ 0.4–0.6 and then induced with 1 mM IPTG for 5 h at 37°C, or with 50 µM IPTG over night at 16°C (in case of flot-1-FL-GST and flot-1-CT-GST). The cells were pelleted and lysed in lysis buffer (50 mM Hepes pH 7.5, 150 mM NaCl, 1 mM EDTA, 5% glycerol, 0.1% NP-40) supplemented with with 100 µg/ml lysozyme, 1 mM PMSF, 1 mM dithiothreitol (DTT) and 1 mM aprotinin, leupeptin and pepstatin. GST proteins from the lysates were allowed to bind to glutathione-sepharose beads (GE Healthcare), washed with PBS and left on the beads for pull-downs.

### His-tagged protein expression and purification

The bacterial expression strain SE1 transformed with FRS2-His was grown and induced as above. The bacteria were lysed in His-lysis buffer (50 mM NaH_2_PO_4,_ 300 mM NaCl, 5% glycerol, 0.1% NP-40) containing 100 µg/ml lysozyme, 1 mM PMSF, 5 mM imidazole, 2 mM β-mercaptoethanol and 1 mM of each of the protease inhibitors (aprotinin, leupeptin, pepstatin). FRS2-His was bound to Ni-NTA agarose beads (Qiagen, Hilden, Germany). The beads were washed with the lysis buffer supplemented with increasing concentrations of imidazole, and FRS2-His was eluted from the beads with lysis buffer containing 250 mM imidazole. To remove excess salt and imidazole, which might interfere with binding in further experiments, the protein was dialysed against the dialysis buffer (50 mM Tris-HCl pH 7.6, 300 mM NaCl, 1 mM EDTA, 1 mM DTT, 20% glycerol) at 4°C over night.

### GST-pulldowns

HeLa or Hep3B cells were lysed for 30 min on ice in lysis buffer (50 mM Tris pH 7.4, 0.15 M NaCl, 2 mM EDTA, 1% NP-40) supplemented with Protease Inhibitor Cocktail (Sigma-Aldrich). Cell lysates were incubated with either GST or GST-tagged proteins immobilized on glutathione-sepharose beads over night at 4°C. The beads were washed three times with 1 ml lysis buffer. The samples were resuspended in loading buffer containing 50 mM DTT, boiled 5 min at 94°C and separated by SDS-PAGE.

### GST pulldown using purified proteins

Direct GST pulldown experiments were performed on ice for 3 h with flicking, using 5 µg of the purified proteins (GST, CAP-GST, FRS2-His). The beads were washed three times with a buffer containing 50 mM Tris-HCl pH 7.5, 150 mM NaCl, 1 mM EDTA, 1 mM DTT, 0.01% Triton X-100, resuspended in 2× SDS sample buffer containing 50 mM DTT, heated for 5 min at 94°C and separated by SDS-PAGE.

### Stable knockdown of flotillins using lentiviruses

Hep3B cells were seeded on a 96-well plate at a density of 32 000 cells/well one day prior to transduction. The cells were infected with MISSION shRNA Lentiviral Transduction Particles (Sigma-Aldrich) with a multiplicity of infection of 5.6. Hexadimethrine bromide (100 µg/ml) was used to increase the efficiency of infection. Knockdown of flot-2 was achieved by using viral clones TRCN0000149396 and TRCN0000150223, whereas for the knockdown of flot-1, clones TRCN0000029310 and TRCN0000029309 were used. Viruses were removed one day after infection. Selection for stable cell lines was started two days post-infection using 2 µg/ml of puromycin.

### Transient knockdown of flot-1 using siRNA oligos

HeLa cells were grown on 12 well plates to a confluency of 80% and transfected with 85 nM siRNA oligonucleotide duplexes targeting flot-1 (Stealth™ siRNA; Invitrogen) using Lipofectamine 2000 (Invitrogen). As a control, an oligo which does not target any human sequence (Stealth™ RNAi Negative Control Duplexes medium GC, Invitrogen) was used. These duplexes have previously been well characterized by us [25], [27] and routinely result in more than 80% knockdown of flot-1 in about 95% of the cells. No off-target effects have been detected so far.

### Growth factor treatment

Hep3B cells were starved 18 h prior to treatment with 100 µM sodium pervanadate for 30 min. HeLa cells were serum starved for 24 h and then treated with 50 ng/ml bFGF (Sigma-Aldrich) and 10 µg/ml heparin.

### Immunofluorescence

Hep3B cells were grown on coverslips and fixed with ice-cold methanol. Unspecific binding was blocked with 1% BSA/PBS, and the cells were labeled with primary antibodies for 1 h and stained with Cy3 and AlexaFluor 488 conjugated secondary antibodies for 45 min. The samples were embedded in Gelmount (Biomeda, Foster City, CA, USA) supplemented with 1,4-diazadicyclo(2,2,2)octane (DABCO, Fluka, Neu-Ulm, Germany). Images were taken with a confocal laser-scanning microscope (Zeiss LSM510 Meta).

### Coimmunoprecipitation

Cells were lysed for 30 min on ice in coimmunoprecipitation buffer (100 mM Tris pH 8.0, 0.15 M NaCl, 1 mM MgCl_2_, 1% Triton X-100), supplemented with Protease Inhibitor Coctail, 60 mM n-octyl-β-D-glucopyranosid (AppliChem) and 1 mM vanadate. Mouse tissues were grinded in liquid nitrogen. 25 mg of powdered frozen tissue were homogenized in a tissue lyzer (Retsch, Germany) in 500 µl coimmunoprecipitation buffer. The lysates were precleared three times with 50 µl Pansorbin beads (Calbiochem, Nottingham, UK). Precipitation was performed using antibody-coupled Protein A Dynabeads (Invitrogen) for 16 h at 4°C. The beads were washed three times with lysis buffer. Precipitated proteins were solubilized in loading buffer containing 50 mM DTT and boiled 5 min at 94°C. Proteins were separated by SDS-PAGE and subjected to immunoblotting with specific antibodies.

### Preparation of detergent insoluble membranes

Hep3B cells were grown in 15 cm dishes, starved overnight and treated or not with 100 µM sodium pervanadate for 30 min. Lipid rafts were prepared using density gradient centrifugation as described [40]. 1.2 ml fractions of the gradients were collected, supplemented with SDS (final concentration 2%) and mixed with 4× loading buffer containing 100 mM DTT and 20% β-mercaptoethanol. The samples were boiled for 5 min at 94°C. Equal volumes were analyzed by SDS-PAGE and Western blotting. In order to detect the ganglioside GM1, 5 µl of the fractions were slot-blotted onto nitrocellulose and incubated with 0.5 µg/ml cholera toxin subunit B (CTX-B; Invitrogen, Karlsruhe, Germany) conjugated with HRP.

### Statistics

Unless otherwise stated, all experiments were performed at least three times. For the statistical analysis, Western blots were quantified by scanning densitometry using Quantity One Software (Biorad, Munich, Germany). Data are shown as mean ± SD. Statistical comparisons between groups were made using one way ANOVA analysis (Bonferoni test) using GraphPad Prism 4 software. Values of *p*<0.05 were considered significant (*) while values of *p*<0.01 and *p*<0.001 were defined very significant (**) and extremely significant (***), respectively.

## Supporting Information

Figure S1 **Expression of the proteins during yeast two-hybrid analysis and analysis of knockdown efficiency of flotillins in the stable Hep3B cells.** (A) Flot-1 and FRS2 domain constructs used for identification of the interacting domains in yeast two-hybrid assay. (B) Yeast lysates were prepared from the transformed cells used for the yeast two-hybrid analysis, and the expression of the fusion proteins was detected. Autoactivation test was done by plating yeast colonies transformed with the bait onto the selective plate containing the substrate of α-galactosidase (–T+α-X-Gal plate). (C) Western blot analysis of knockdown efficiency of flot-1 and flot-2. GAPDH was used as an equal loading control. (D) Immunofluorescence staining of endogenous flotillins in control (left column), flot-2 knockdown (upper right) and flot-1 knockdown (lower right) cells. Scale bars 10 µm.
(TIF)
