# Supplementary figures and images for: Molecular Networks in FGF Signaling: Flotillin-1 and Cbl-Associated Protein Compete for the Binding to Fibroblast Growth Factor Receptor Substrate 2
Source: PLoS One. 2012 Jan 3;7(1):e29739. doi: 10.1371/journal.pone.0029739 (PMC3250484; doi:10.1371/journal.pone.0029739)

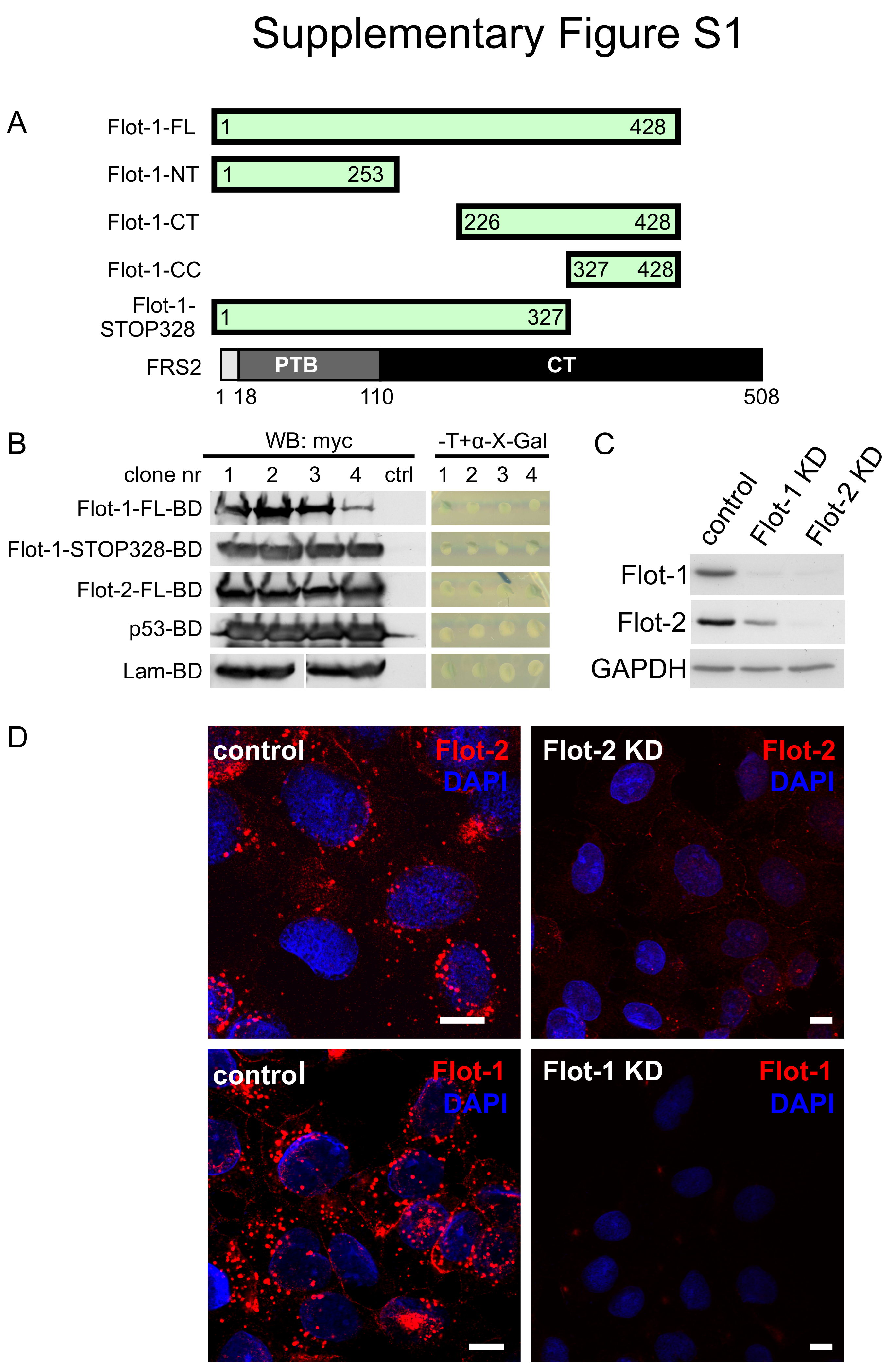

Supplement: Figure S1 — Expression of the proteins during yeast two-hybrid analysis and analysis of knockdown efficiency of flotillins in the stable Hep3B cells. (A) Flot-1 and FRS2 domain constructs used for identification of the interacting domains in yeast two-hybrid assay. (B) Yeast lysates were prepared from the transformed cells used for the yeast two-hybrid analysis, and the expression of the fusion proteins was detected. Autoactivation test was done by plating yeast colonies transformed with the bait onto the selective plate containing the substrate of α-galactosidase (–T+α-X-Gal plate). (C) Western blot analysis of knockdown efficiency of flot-1 and flot-2. GAPDH was used as an equal loading control. (D) Immunofluorescence staining of endogenous flotillins in control (left column), flot-2 knockdown (upper right) and flot-1 knockdown (lower right) cells. Scale bars 10 µm. (TIF) [file pone.0029739.s001.tif]
